# Characterization and Detection of *ϵ*-Berge-Zhukovskii Equilibria

**DOI:** 10.1371/journal.pone.0131983

**Published:** 2015-07-15

**Authors:** Rodica Ioana Lung, Mihai Suciu, Noémi Gaskó, D. Dumitrescu

**Affiliations:** 1 Faculty of Economics and Business Administration, Babeş-Bolyai University, Cluj-Napoca, Romania; 2 Faculty of Mathematics and Computer Science, Babeş-Bolyai University, Cluj-Napoca, Romania; Nankai University, CHINA

## Abstract

The Berge-Zhukovskii equilibrium is an alternate solution concept in non-cooperative game theory that formalizes cooperation in a noncooperative setting. In this paper, the *ϵ*-Berge-Zhukovskii equilibrium is introduced and characterized by using a generative relation. The generative relation also provides a solution to the problem of computing the *ϵ*-Berge-Zhukovskii equilibrium for large games, by using evolutionary algorithms. Numerical examples illustrate the approach and provide a possible application for this equilibrium concept.

## Introduction

Non-cooperative game theory models players that make decisions independently based on their own interests. Different equilibrium concepts are used to provide decision makers with an overview over possible outcomes of the game. The most popular equilibrium concept is the Nash equilibrium [1]—a game situation from which no player has an incentive to unilaterally deviate. A more general equilibrium concept based on the notion of equilibrium for a partition with respect to a coalition was proposed by Berge [2]. Zhukovskii [3] formalized a particularization of the general Berge equilibrium and introduced it as an alternate solution concept that is complementary to Nash and more suited for games where the Nash equilibrium (NE) has no practical value (e.g. trust games [4]). The equilibrium defined by Zhukovskii is referred to as Berge equilibrium in the sense of Zhukovskii [5, 6], or simply as the Berge equilibrium [7, 8], or as the Berge-Zhukovskii (BZ) equilibrium.

The Berge-Zhukovskii equilibrium is a situation in which the payoff of each player cannot decrease considering all possible deviations of *all* the other players, implying that players are other-regarding, while in NE players are self-regarding. BZ may also be expressed as the situation of the game in which each player has a maximum payoff under all possible deviations of all other players.

Existence theorems and characterizations for BZ equilibrium can be found in [6, 9–11] and [5]. A conclusion that can be drawn from these studies is that the BZ does not exist in all games. A connection between the BZ and the Nash equilibrium for several two person games and a method to find the BZ in *n*-player games is presented in [7]. To the best of our knowledge the first computational intelligence approach aimed to directly compute the Berge-Zhukovskii equilibrium for large games, based on a generative relation, is described in [12].

The *ϵ*-Nash equilibrium, introduced by Radner [13], can be viewed as a weakening of the strict rationality—in this case it is enough to be “near” to the Nash equilibrium, or to approximate the Nash equilibrium. *ϵ* can be interpreted in several ways: measuring the uncertainty of selecting a strategy, measuring the supplementary cost of attending the equilibrium strategy, or as a perturbation of the players rationality [14].

The *ϵ*-Berge-Zhukovskii equilibrium is introduced in a similar manner. The intuition behind it is the same as in the case of the *ϵ*-Nash equilibrium: *ϵ* allows for a perturbation in players strategies. Thus, we may consider also that *ϵ*-BZ provides the flexibility needed for solving complex, real, games. Moreover, *ϵ*-BZ is further characterized by using a generative relation in a similar manner with [12].

A generative relation for a game equilibrium is a binary relation defined on strategy profiles such that the set of strategy profiles non-dominated with respect to that relation is identical with the set of that equilibria of the game (non-dominated strategies are those for which there does not exists ‘better’ ones with respect to the generative relation) [15, 16]. The generative relation defined for *ϵ*-BZ is used to guide the search of an evolutionary optimization algorithm in order to compute the *ϵ*-BZ of a game.

## Methods

The Methods part of the paper theoretically describes the BZ and *ϵ*-BZ equilibria, their characterization, and the proposed computational approach.

### *ϵ*-Berge-Zhukovskii equilibrium

A finite strategic non-cooperative game is a system

$$\begin{array}{c} G=(\left(N,S_{i},u_{i}\right),i=1,...,n), \end{array}$$

where:

- *N* represents the set of players, and *n* is the number of players;
- *S*_*i*_ is the set of actions available to player *i* ∈ *N*, and
  $$\begin{array}{c} S=S_{1}\times S_{2}\times ...\times S_{n} \end{array}$$
  is the set of all possible situations of the game; *s* = (*s*_1_, …*s*_*n*_) ∈ *S* is a strategy (or strategy profile) of the game;
- for each player *i* ∈ *N*, *u*_*i*_ : *S* → ℝ represents the payoff function of player *i*.

For *i* ∈ *N*, and *s*, *q* ∈ *S*, we denote by *S*_−*i*_ = *S*_1_ × … × *S*_*i*−1_ × *S*_*i*+1_ × … × *S*_*n*_, by *s*_−*i*_ = (*s*_1_, …, *s*_*i*−1_, *s*_*i*+1_, …, *s*_*n*_), and $\left(q_{i}^{*},s_{-i}^{})=(s_{1},s_{2},...,q_{i}^{*},...,s_{n}\right)$; for *R* ⊂ *N*, −*R* = {*i* ∈ *N*∣*i* ∉ *R*}, *S*_*R*_ = ∏_*i* ∈ *R*_ *S*_*i*_, *S*_−*R*_ = ∏_*j* ∈ −*R*_ *S*_*j*_, and (*q*_−*R*_, *s*_*R*_) the strategy profile in which players *i* ∈ *R* choose *s*_*i*_ and players *j* ∈ −*R* choose *q*_*j*_.

The general Berge equilibrium is formally defined as follows:

**Definition 1 (Berge equilibrium)** *Denote by* *P* = {*P*_*t*_}_*t* ∈ *M*_ *a partition of* *N* *and* *R* = {*R*_*t*_}_*t* ∈ *M*_ *be a set of subsets of* *N*. *A strategy profile* s* ∈ *S* *is an equilibrium strategy for the partition* *P* *with respect to the set* *R*, *or simply a Berge equilibrium strategy, if and only if the condition*

$$\begin{array}{c} u_{p_{m}}\left(s^{*}\right)\geq u_{p_{m}}\left(s_{-R_{m}}^{*},s_{R_{m}}^{}\right) \end{array}$$

*holds for each given* *m* ∈ *M*, *any* *p*_*m*_ ∈ *P*_*m*_ *and* *s*_*R*_*m*__ ∈ *S*_*R*_*m*__.

If we consider *P* = {{*i*}:*i* ∈ *N*} and *R* = {{*i*}:*i* ∈ *N*} in Definition 1, it is clear that the Nash equilibrium is a Berge equilibrium for *P* relative to *R*, so any Nash equilibrium is a Berge equilibrium. Obviously, the converse is not true—not all Berge equilibria are Nash equilibria.

On the other hand, if we consider that each class *P*_*i*_ of the partition *P* consists from player *i*, and each set of *R*_*i*_ is the set *N* of players except *i*, i.e. *M* = *N*, *P*_*i*_ = {*i*}, and *R*_*i*_ = *N*−{*i*}, ∀*i* ∈ *N*, we obtain the Berge-Zhukovskii equilibrium as proposed by Zhukovskii in [3]. Formally we write:

**Definition 2 (Berge-Zhukovskii)** *A strategy profile* s* ∈ *S* *is a Berge-Zhukovskii equilibrium if the inequality*

$$\begin{array}{c} u_{i}\left(s^{*}\right)\geq u_{i}\left(s_{i}^{*},s_{-i}^{}\right) \end{array}$$

*holds for each player* *i* = 1, …, *n*, *and all* *s*_−*i*_ ∈ *S*_−*i*_.

Playing in Berge-Zhukovskii sense can be interpreted in two ways: (a) each player is ensuring a payoff that will not decrease under any deviations of the other players and (b) each player maximizes the payoff of the other players. Thus, this equilibrium concept can be interpreted as capturing cooperation in a non-cooperative game.

**Example 1** *Let us consider the Prisoner’s Dilemma (PD) game presented in Table 1*.

**Table 1:** Payoffs for a Prisoner’s Dilemma game.

Player 2			
		Cooperate	Defect
Player 1	Cooperate	(2, 2)	(0, 3)
	Defect	(3, 0)	(1, 1)

*Let us analyze the game: the strategy* $\left(s_{1}^{*},s_{2}^{*}\right)$ *is a NE if the following inequalities hold*: $u_{1}(s_{1}^{*},s_{2}^{*})\geq u_{1}(s_{1},s_{2}^{*}),$ $u_{2}(s_{1}^{*},s_{2}^{*})\geq u_{2}(s_{1}^{*},s_{2}),$ ∀*s*_1_, *s*_2_ ∈ *S*, *and* $\left(s_{1}^{*},s_{2}^{*}\right)$ *is a BZ if*: $u_{1}(s_{1}^{*},s_{2}^{*})\geq u_{1}(s_{1}^{*},s_{2}),$ $u_{2}(s_{1}^{*},s_{2}^{*})\geq u_{2}(s_{1},s_{2}^{*}),$ ∀*s*_1_, *s*_2_ ∈ *S*.

*The Nash equilibrium of this game*, *(Defect, Defect)*, *does not ensure the highest possible payoff for both players. Conversely, the Berge-Zhukovskii equilibrium of the game is* (Cooperate, Cooperate), *which leads to better payoffs for both players and induces cooperation*.

Inspired by the notion of *ϵ*-Nash equilibrium, the *ϵ*-Berge-Zhukovskii (*ϵ*-BZ) equilibrium is introduced in what follows. The new equilibrium concept provides flexibility to the standard Berge-Zhukovskii equilibrium.

**Definition 3 (*ϵ*-Berge-Zhukovskii equilibrium)** *A strategy profile* s* ∈ *S* *is an* *ϵ*-*Berge-Zhukovskii equilibrium if the inequality*

$$\begin{array}{c} u_{i}\left(s^{*}\right)\geq u_{i}\left(s_{i}^{*},s_{-i}^{}\right)-\epsilon ,\epsilon \geq 0 \end{array}$$

*holds for each player* *i* = 1, …, *n*, *and* ∀ *s*_−*i*_ ∈ *S*_−*i*_.

**Remark 1** *If* *ϵ* = 0 *the* *ϵ*-*BZ is actually the Berge-Zhukovskii equilibrium*.

We denote by *BZ*_*ϵ*_ the set of all *ϵ*- Berge-Zhukovskii equilibria of the game.

**Example 2** *Let us consider the two-person continuous game* [17], *having the following payoff functions*:

$$\begin{array}{ccc} & & u_{1}\left(s_{1},s_{2}\right)=-s_{1}^{2}-s_{1}+s_{2},\\ & & u_{2}\left(s_{1},s_{2}\right)=2s_{1}^{2}+3s_{1}-s_{2}^{2}-3s_{2},\\ & & s_{i}\in \left[-2,1\right],i=1,2. \end{array}$$

*The Berge-Zhukovskii equilibrium of the game is* (1, 1) *with the corresponding payoffs* (−1, 1). *If we consider* *ϵ* = 0.1, *it is easy to see that the strategy profile* (0.999,0.999) *is an* *ϵ*-*BZ of the game, because the following inequalities hold for every* *s*_1_, *s*_2_ ∈ [−2, 1]: *u*_1_(0.999,0.999) ≥ *u*_1_(0.999, *s*_2_)−0.1, *and* *u*_2_(0.999, 0.999) ≥ *u*_2_(*s*_1_,0.999)−0.1.

*The strategy profile* (0.999, 0.999) *is just one* *ϵ*-*BZ from the infinitely number of* *ϵ*-*BZ equilibria for this game*.

### Characterization of *ϵ*-Berge-Zhukovskii equilibrium

Generative relations [15, 18] characterize a certain equilibrium and can be used for fitness assignment purposes within optimization heuristics to guide their search towards a desired equilibrium type. The first generative relation was introduced for Nash equilibria detection [15]. A generative relation for the detection of Berge-Zhukovskii equilibrium was introduced in [12], but without a formal proof.

Consider two strategy profiles *s* and *q* from *S* for game *G*. We denote by *b*_*ϵ*_(*s*, *q*) the number of players that benefit (with a deviation of *ϵ*) by remaining to the initial strategy *s*, while all other players are switching their strategies to *q*, if they are all different from *s*. Thus, *b*_*ϵ*_(*s*, *q*) is:

$$\begin{array}{c} b_{\epsilon }\left(s,q\right)=card\left\{i\in N,u_{i}\left(s\right)<u_{i}\left(s_{i}^{},q_{-i}\right)+\epsilon ,s_{-i}\neq q_{-i}\right\}, \end{array}$$

where *card*{*M*} denotes the cardinality of the set *M* and *s*_−*i*_ ≠ *q*_−*i*_ ⇔ *s*_*j*_ ≠ *q*_*j*_ for all *j* = 1, …, *n*, *j* ≠ *i*.

The intuition behind the construction of *b*_*ϵ*_ is that in the search for *ϵ*-BZ we minimize the number of players whose payoff would increase when all the others switch to different strategies. Thus, *b*_*ϵ*_ can be used to compare two strategy profiles.

**Definition 4** *Let* *s*, *q* ∈ *S*. *We say the strategy* *s* *is better than strategy* *q* *or that* *s* ***dominates*** *q* *with respect to* *ϵ*-*Berge-Zhukovskii equilibrium, and we write* *s* ≺_*B*_*ϵ*__ *q*, *if and only if the inequality*

$$\begin{array}{c} b_{\epsilon }\left(s,q\right)<b_{\epsilon }\left(q,s\right) \end{array}$$

*holds, i.e. there are less players that would benefit when all the others change their strategies from* *s* to *q* *than from* *q* to *s*.

**Remark 2** *If* *b*_*ϵ*_(*s*, *q*) = *b*_*ϵ*_(*q*, *s*), *we consider* *s* *and* *q* *to be* *indifferent* *to each other with respect to the* ≺_*B*_*ϵ*__ *relation*.

**Definition 5** *The strategy profile* s* ∈ *S* *is an* *ϵ*-*Berge-Zhukovskii **non-dominated strategy*** *(BZN*_*ϵ*_ *), if and only if there is no strategy* *s* ∈ *S*, *s* ≠ s* *such that* *s* *dominates* s* *with respect to ≺*_*B*_*ϵ*__, *i.e.*

$$\begin{array}{c} ∄s\in S,s{≺}_{B_{\epsilon }}s^{*}. \end{array}$$

In the following we will show that relation ≺_*B*_*ϵ*__ is a generative relation of the *ϵ*-Berge-Zhukovskii equilibrium, i.e. the set of non-dominated strategies with respect to the relation ≺_*B*_*ϵ*__ equals the set of *ϵ*-Berge-Zhukovskii equilibria of the game.

**Proposition 1** *If a strategy profile* s* ∈ *S* *is an* *ϵ*- *Berge-Zhukovskii equilibrium, then the equality*

$$\begin{array}{c} b_{\epsilon }\left(s^{*},s\right)=0 \end{array}$$

*holds, for all* *s* ∈ *S*.

**Proof 1** *Let* s* ∈ *BZ*_*ϵ*_. *Suppose there exists a strategy profile* *s* ∈ *S*, *such that* *b*_*ϵ*_(s*, *s*) = *w*, *w* > 0. *Therefore there exists* *i* ∈ *N*, *such that*

$$\begin{array}{c} u_{i}\left(s_{i}^{*},s_{-i}^{}\right)+\epsilon >u_{i}\left(s^{*}\right). \end{array}$$

*This contradicts the definition of the* *ϵ*- *Berge-Zhukovskii equilibrium. Hence* *b*_*ϵ*_(s*, *s*) = 0.

**Proposition 2** *All* *ϵ*-*Berge-Zhukovskii equilibria are* *ϵ*-*Berge-Zhukovskii non-dominated strategies and all* *ϵ*-*Berge-Zhukovskii non-dominated strategies are* *ϵ*-*Berge-Zhukovskii equilibria*:

$$\begin{array}{c} BZN_{\epsilon }=BZ_{\epsilon }. \end{array}$$

**Proof 2** *First we prove the following: All* *ϵ*-*BZ equilibrium strategies are* *ϵ*-*Berge-Zhukovskii non-dominated strategies, i.e.* *BZ*_*ϵ*_ ⊆ *BZN*_*ϵ*_.

*Let* s* ∈ *BZ*_*ϵ*_. *Suppose* s* *is dominated. Therefore there exists a strategy profile* *s* ∈ *S* *dominating* s*:

$$\begin{array}{c} s{≺}_{B_{\epsilon }}s^{*}. \end{array}$$

*From definition of the relation* ≺_*B*_*ϵ*__ *we have*

$$\begin{array}{c} b_{\epsilon }\left(s,s^{*}\right)<b_{\epsilon }\left(s^{*},s\right). \end{array}$$

*As* s* *is an* *ϵ*-*Berge-Zhukovskii equilibrium from Prop. 1 it follows that*

$$\begin{array}{c} b_{\epsilon }\left(s^{*},s\right)=0. \end{array}$$

*Thus we have*

$$\begin{array}{c} b_{\epsilon }\left(s,s^{*}\right)<0. \end{array}$$

*But this is not possible, because* *b*_*ϵ*_(*s*, s*) *denotes the cardinality of a set*. *Therefore* s* *is from* *BZN*_*ϵ*_ *(i.e. non-dominated)*.

*Next we prove the following: All* *ϵ*-*Berge-Zhukovskii non-dominated strategies are* *ϵ*-*BZ equilibrium strategies, i.e.*

$$\begin{array}{c} BZN_{\epsilon }\subseteq BZ_{\epsilon }. \end{array}$$

*Let us consider* s* ∈ *BZN*_*ϵ*_ (s* *is a non-dominated strategy profile) and suppose that* s* ∉ *BZ*_*ϵ*_.

*If* s* ∉ *BZ*_*ϵ*_ ⇒ ∃*s*_−*i*_ *such that*

$$\begin{array}{c} u_{i}\left(s_{i}^{*}\right)<u_{i}\left(s_{i}^{*},s_{-i}\right)+\epsilon , \end{array}$$ (1)

*and* $s_{-i}^{*}\neq s_{-i}.$

*Let us denote by* *q* *the strategy profile* $(s_{i}^{*},s_{-i}).$

*We have*:

$$\begin{array}{c} b_{\epsilon }\left(s^{*},q\right)=card\left\{j\in N,u_{j}\left(s^{*}\right)<u_{j}\left(s_{j}^{*},q_{-j}\right)+\epsilon ,s_{-j}^{*}\neq q_{-j}\right\}. \end{array}$$

*But for all* *j* ≠ *i* *we have* $s_{i}^{*}=q_{i}$ *so* $s_{-j}^{*}\neq q_{-j}$ *does not hold, and for* *j* = *i* *relation (1) holds, therefore* *b*_*ϵ*_(s*, *q*) = 1.

*On the other hand*

$$\begin{array}{c} b_{\epsilon }\left(q,s^{*}\right)=card\left\{j\in N,u_{j}\left(q\right)<u_{j}\left(q_{j},s_{-j}^{*}\right)+\epsilon ,q_{-j}\neq s_{-j}^{*}\right\}. \end{array}$$

*If* *i* ≠ *j* $s_{i}^{*}=q_{i}$ *so* $q_{-j}\neq s_{-j}^{*}$ *does not hold*.

*If* *i* = *j* *we have* $(q_{i},s_{-i}^{*})=s^{*},$ *therefore* $u_{i}(q)<u_{i}(q_{i},s_{-i}^{*}),$ *if and only if*

$$\begin{array}{c} u_{i}\left(q\right)<u_{i}\left(s^{*}\right)+\epsilon \end{array}$$

*if*

$$\begin{array}{c} u_{i}\left(s_{i}^{*},s_{-i}\right)<u_{i}\left(s^{*}\right)+\epsilon \end{array}$$

*which would contradict relation (1). Therefore* *b*_*ϵ*_(*q*, s*) = 0.

*We have that* *b*_*ϵ*_(s*, *q*) > *b*_*ϵ*_(*q*, s*), *which contradicts the assumption of non-domination, therefore* s* *is an* *ϵ*-*BZ equilibrium*.

### Equilibrium detection

Evolutionary algorithms (EAs) [19, 20] are search and optimization techniques that use nature inspired operators to provide approximate solutions to complex problems that cannot be tackled by classical methods. EAs are supposed to be adaptive and scalable, i.e. easily adapted to different types of problems and able to cope with dimensions increases in the search space.

Within EAs, a population of potential solutions called individuals, randomly generated at the beginning of the search, is evolved for several generations by using selection and variation operators, until a satisfactory solution is found. The process is based on the “survival of the fitness” paradigm: a fitness function is used to evaluate individuals which compete for survival during the selection process while new ones are created each generation by using the variation operators. Thus, the fitness value is used to compare and select individuals. Comparisons are performed using a relation defined on the set of fitness values. For example, uni-objective optimization methods use the simple order relation in ℝ, while some multi-objective optimization methods use the Pareto domination relation [21].

Our assumption is that an EA can be adapted to compute the *ϵ*-BZ of a game if the generative relation ≺_*B*_*ϵ*__ is used during the selection process to direct the search towards the *ϵ*-BZ non-dominated solutions. Thus, when two individuals are compared, the one that *ϵ*-BZ dominates the other will be chosen during the selection process.

The performance of an EA is usually validated by means of numerical experiments. In order to test our assumption we will consider two games, one with a known BZ and corresponding set of *ϵ*-BZ, and another one, constructed by us as a potential practical application for the Berge-Zhukovskii equilibrium concept, derived from the network community structure detection problem.

#### Voluntary contribution mechanism

The Voluntary Contribution Mechanism (*VCM*) is a good example that illustrates the fact that people may not be totally self-interested, and that they may spend time for the common good (based on theoretical studies [22] and experiments [23, 24] concerning player’s behavior). A model of the *VCM* is described as having the following payoff functions:

$$\begin{array}{c} u_{i}\left(s\right)=10-s_{i}+0.4\sum_{i=1,n}s_{i},s_{i}\in \left[0,10\right],i=1,...,n. \end{array}$$

In this game the Berge-Zhukovskii equilibrium is achieved when all players play strategy 10, which means they spend everything for the public good.

If we consider the three players version of the game $\left(s_{1}^{*},s_{2}^{*},s_{3}^{*}\right)$ is an *ϵ*-BZ equilibrium if the following inequalities hold:

$u_{1}(s_{1}^{*},s_{2}^{*},s_{3}^{*})\geq u_{1}(s_{1}^{*},s_{2},s_{3})-\epsilon ,$

$u_{2}(s_{1}^{*},s_{2}^{*},s_{3}^{*})\geq u_{2}(s_{1},s_{2}^{*},s_{3})-\epsilon ,$

$u_{3}(s_{1}^{*},s_{2}^{*},s_{3}^{*})\geq u_{3}(s_{1},s_{2},s_{3}^{*})-\epsilon ,$ ∀*s*_1_, *s*_2_, *s*_3_ ∈ [0, 10].

For example, if we take *ϵ* = 0.1 the strategy profile (9.99, 9.99, 9.99) is an *ϵ*-BZ of the game.

This game will be approached by using a well known evolutionary multiobjective algorithm, called the Nondominated Sorting Genetic Algorithm II (NSGA-II) [25]. NSGA-II is an evolutionary algorithm that uses the Pareto domination relation to guide the search towards the Pareto optimal solutions, with source code publicly available (http://www.iitk.ac.in/kangal/codes.shtml last accessed 12/3/2015). The only change made to the original source code was to substitute the Pareto domination test with the ≺_*B*_*ϵ*__ relation. The adapted version was called BZ-NSGA-II.

#### Community structure detection in complex networks

Probably among the reasons why the Berge-Zhukovskii equilibrium has not been used greatly is the lack of a practical application to illustrate its properties, together with an effective computational method for computing it.

In recent years, the part of network analysis concerned with finding community structures in complex networks has drawn a lot of attention due to its large applicability in various fields (social sciences, economics, politics, physics, biology, etc.) [26]. The problem consists in finding “communities” of nodes that are highly connected to each other and sparsely connected to nodes outside [27].

In the following, we propose a novel application that considers the use of the Berge-Zhukovskii equilibrium within a community structure detection algorithm for complex networks.

Consider a network (*V*, *E*), where *V* is a list of nodes and *E* is the list of edges containing pairs of nodes linked to each other, and the game Γ(*V*, *C*, *U*)—first proposed in [28]—where

- *V* is the set of players represented by network nodes, *V* = {1, 2, …, *n*};
- *C*_*i*_ is the set of strategies available to each player *i*, and *C* = *C*_1_ × … × *C*_*n*_; *C*_*i*_ contains the set of possible communities *i* can choose from; an element *c* ∈ *C* is a strategy profile of the game, *c* = (*c*_1_, *c*_2_, …*c*_*n*_), and *c*_*i*_ is the community of player *i*;
- *U* = (*u*_1_, *u*_2_, …, *u*_*n*_) is the payoff function, with *u*_*i*_:*C* → ℝ, ∀*i* ∈ *V*; *u*_*i*_ is computed as the node fitness defined in [29]:
  $$\begin{array}{c} u_{i}\left(c_{1},...,c_{n}\right)=f\left(c_{i}\cup \left\{i\right\}\right)-f\left(c_{i}\setminus \left\{i\right\}\right) \end{array}$$ (2)
  where *c*_*i*_∪{*i*} is the community *c*_*i*_ with node *i* included in it, *c*\{*i*} is the community *c*_*i*_ without node *i*, and *f*(*c*) is the community fitness computed as:
  $$\begin{array}{c} f\left(c\right)=\frac{k_{in}}{{\left(k_{in}+k_{out}\right)}^{\alpha }}. \end{array}$$ (3)
  In Eq (3) *k*_*in*_ is the double of internal links of community *c*, *k*_*out*_ is the number of links connecting nodes from *c* with nodes from outside of *c*, and *α* is a parameter related to the size of the community [30].

In [28, 31] it was assumed that the Nash equilibrium of this game—the network partition such that no node can improve its payoff by unilateral deviation—corresponds to a correct cover, assumption supported by successful numerical experiments.

However, the BZ of this game is a network partition that ensures for every node a payoff that would not decrease under any changes made by any of the other nodes. While the existence of the BZ is to be analyzed, the potential of considering the BZ as a solution for the community structure problem can be preliminary tested by using an evolutionary approach similar to the one used in Example 1. As NSGA-II has known limitations for many-objective problems (more than 5–6 objectives), and most networks will have more nodes, we will use an extremal optimization algorithm called Berge Extremal Optimization (BEO), adapted from the Nash Extremal Optimization in [32] which reports very good results on games with large number of players. NEO was previously adapted for the dynamic community structure detection problem in [31].

BEO is an adaptation of the NEO in [32] for game Γ with the following changes:

- the Nash ascendancy test is replaced with the ≺_*B*_*ϵ*__ domination relation; to refine the search, the value of *ϵ* is linearly decreased from an initial value to 0.
- within NEO, each iteration, the player with the worst payoff is assigned a different strategy; within BEO a number *k* of the players having the worse payoffs will have their strategies changes; for the community detection game *k* linearly decreases from 10% of the number of nodes (players) to 1 during the first half of the search, and remains 1 until the end.
- NEO evolves a pair of configurations; BEO uses a population of pairs of configuration that evolve independently; each pair searches for a cover containing a certain number of communities, this number is set between a minimum and a maximum possible number of communities.

## Results and Discussion

In this section numerical results obtained for the two experiments will be presented and discussed.

### Experiment 1—Voluntary contribution mechanism

For all the tests a population of 150 individuals is used for 150 generations of the BZ-NSGA-II. As for the variation operators we use a distribution indexes for mutation and crossover *η*_*m*_ = 20 and *η*_*c*_ = 20 ([25]).

**Fig 1:**
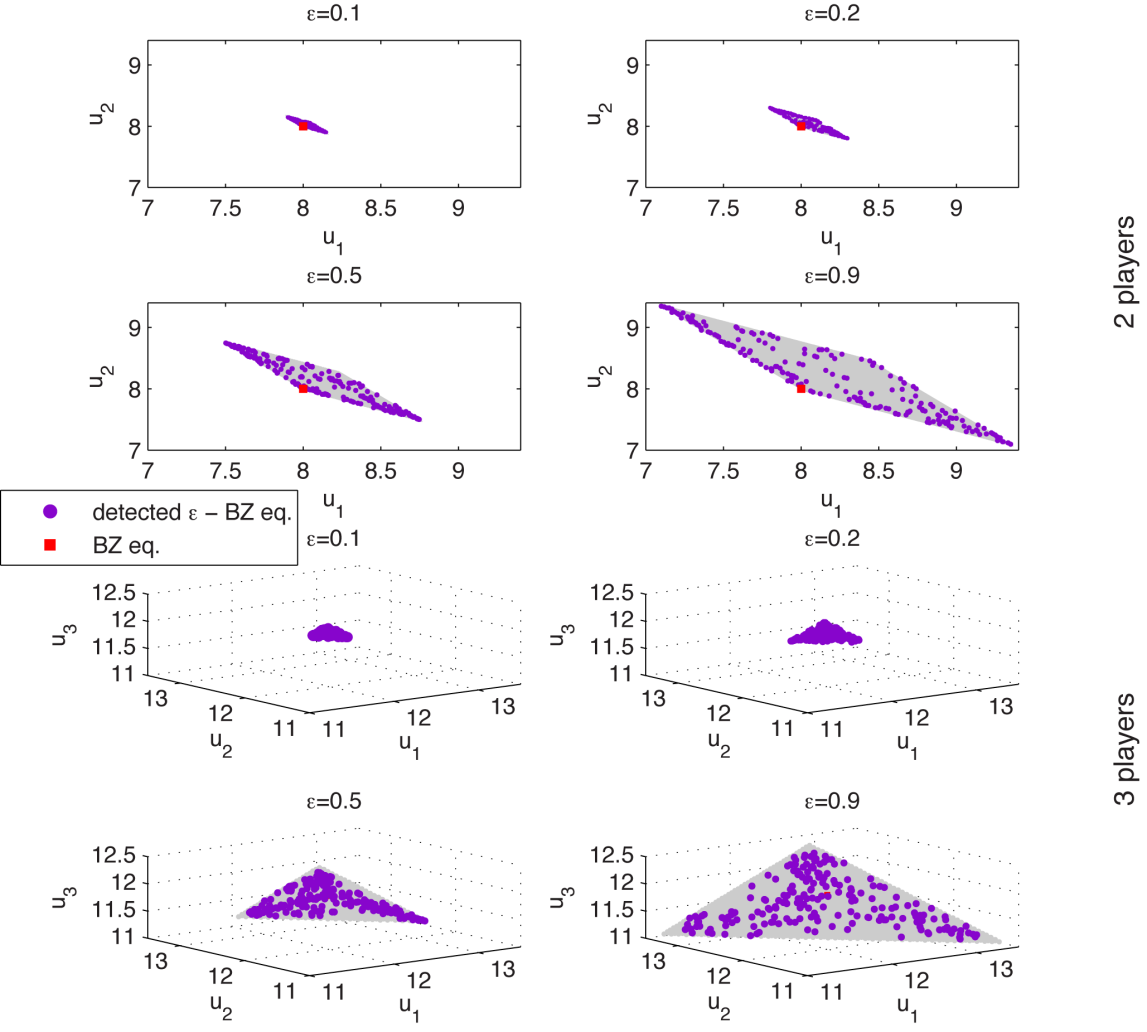
Payoffs for Voluntary Contribution Mechanism for 2 and 3 players and different values of *ϵ* ∈ {0.1,0.2,0.5,0.9}. The grey area represents the theoretical front, covered by the solutions detected by BZ-NSGA-II.

The obtained results are illustrated in Fig 1 in the following manner: for two and three players the payoffs space is represented by assigning axes to the payoff of each player and representing payoffs as points in the two, and three dimensional spaces respectively. In each graphic the set of *ϵ*-BZ is represented with gray color.

As it is natural to expect, by increasing the value of *ϵ* the number of *ϵ*-Berge-Zhukovskii equilibria also increases by covering a region that includes the Berge-Zhukovskii equilibrium in the payoffs space. In fact, for *ϵ* = 0 the Berge-Zhukovskii equilibrium is obtained.

Numerical experiments presented here illustrate that BZ-NSGA-II is able to find a good approximation of the *ϵ*-Berge-Zhukovskii set for different values of *ϵ* and for different number of players for the voluntary contribution mechanism game.

### Experiment 2—Community structure detection in complex networks

To test the potential use of BZ for the community detection problem we used both synthetic and real-world benchmarks with known community structure and compared the results with those provided by other state-of-art methods from literature.

#### Synthetic networks

The well known GN and LFR benchmarks [27] with 128 nodes are considered. The GN network are characterized by *z*_*out*_, the number of links a node has outside its community (*z*_*out*_ ∈ {1, …, 8} out of the node degree of 16) and the LFR by *μ*, the ratio between the number of links a node has within its community and its total degree (*μ* ∈ {0.1, …, 0.5}). A *z*_*out*_ = 8 value corresponds to *μ* = 0.5. We consider 8 sets for GN and 6 sets for LFR, *z*_*out*_ ∈ {1, …, 8} and *μ* ∈ {0.1, …, 0.5}, respectively. Each set consists of 30 networks (generated using the code available at https://sites.google.com/site/ andrealancichinetti/software).

#### Real world networks

Four real-world datasets having known community structure are used: the bottle-nose *dolphin* network [33], the *football* network [34], the Zachary *karate* club network [35], and the *books* about US politics network (http://www.orgnet.com, last accessed 9/3/2015).

#### Comparisons with other methods

BEO results are compared with those reported by the following state-of-art algorithms: *OSLOM* [36], *Infomap* [37], and Modularity optimization [38] (By using the source code available at https://sites.google.com/site/andrealancichinetti/software with parameters indicated in each paper.).

#### Performance evaluation

The Normalized Mutual Information (NMI) proposed in [30] is used to compare the results provided by search methods with the real community structure of a network. A NMI equal to 1 indicates that the real cover has been identified; between two NMI values, the cover corresponding to the higher value is considered to be better than the other one.

To assess the significance of the results a Wilcoxon sum-rank test is used to compare pairs of results. For each *μ* or *z*_*out*_ value, the results obtained for the 30 networks are compared, while for the real-world networks we run each algorithm 30 times.

#### BEO parameter settings

BEO uses the following parameters: population size 30, maximum number of generations 700 for the synthetic networks with *z*_*out*_ ≤ 7 and *μ* ≤ 0.4, and 10000 generations for the rest; *ϵ* ∈ {0,10^−1^, 10^−2^, 10^−3^, 10^−4^, 10^−5^}.

#### Results and discussion

**Fig 2:**
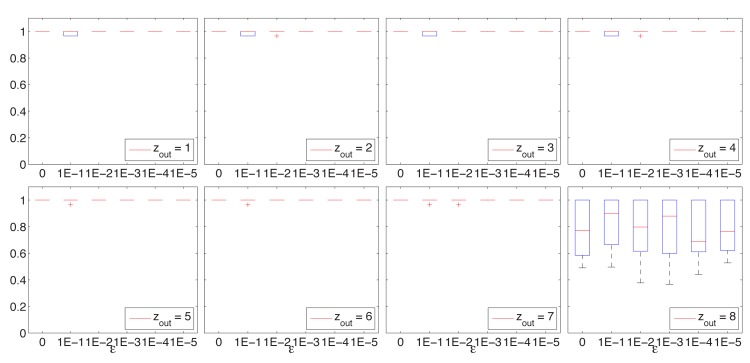
BEO. NMI box-plots for GN networks for *z*_*out*_ ∈ {1, 2, …, 8} and *ϵ* ∈ {0, 10^−1^, 10^−2^, 10^−3^, 10^−4^, 10^−5^}.

**Fig 3:**
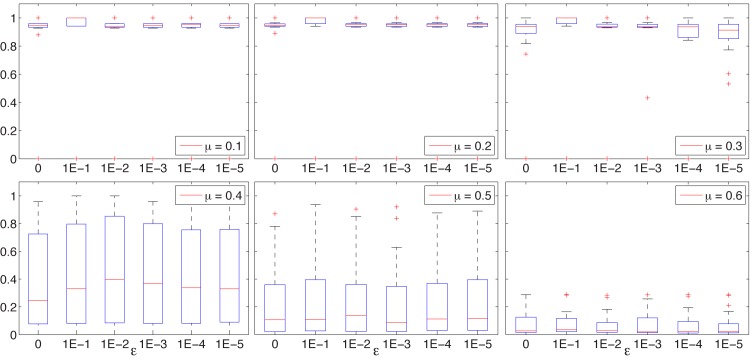
BEO. NMI box-plots for LFR S networks, *μ* ∈ {0.1, 0.2, …, 0.6} and with *ϵ* ∈ {0, 10^−1^, 10^−2^, 10^−3^, 10^−4^, 10^−5^}.

**Fig 4:**
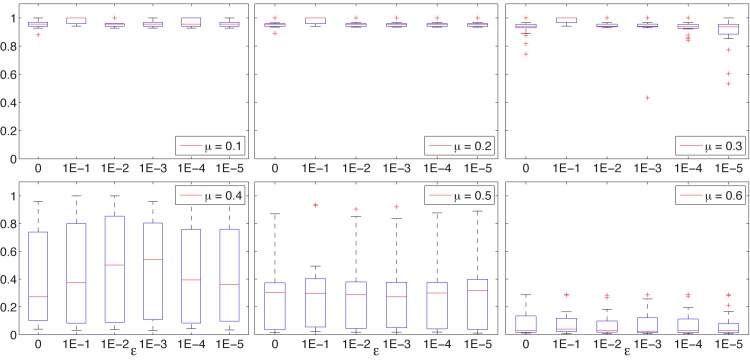
BEO. NMI box-plots for LFR B networks, *μ* ∈ {0.1, 0.2, …, 0.6} and with *ϵ* ∈ {0, 10^−1^, 10^−2^, 10^−3^, 10^−4^, 10^−5^}.

The results obtained for the synthetic networks for various values of *ϵ* are represented as boxplots in Figs 2, 3, and 4.

For the GN sets with *z*_*out*_ ≤ 7, BEO identified the correct cover in almost all instances, except some runs for *ϵ* ∈ {0.1,0.01} indicating that these values may be to high to consider and that they may hinder the search. For *z*_*out*_ = 8 the results are very good considering that the community structure is more difficult to identify as each node has an equal number of links within and without its community. Statistical comparisons of the results show that the values obtained with *ϵ* = 0.001 are better than most of the others, and not worse than any of the others.

The results obtained for the LFR networks, which are considered more difficult and closer to real world networks, are almost similar. For the LFR S sets with *μ* ≤ 0.4, BEO is capable to identify the correct structure with NMI values close to 1 and some outliers, and for *μ* = 0.5 the results resemble the results obtained for GN *z*_*out*_ = 8. For the LFR B sets, the correct cover is easily identified for *μ* ≤ 0.3, with average NMI values decreasing with the increase of *μ*.

**Fig 5:**
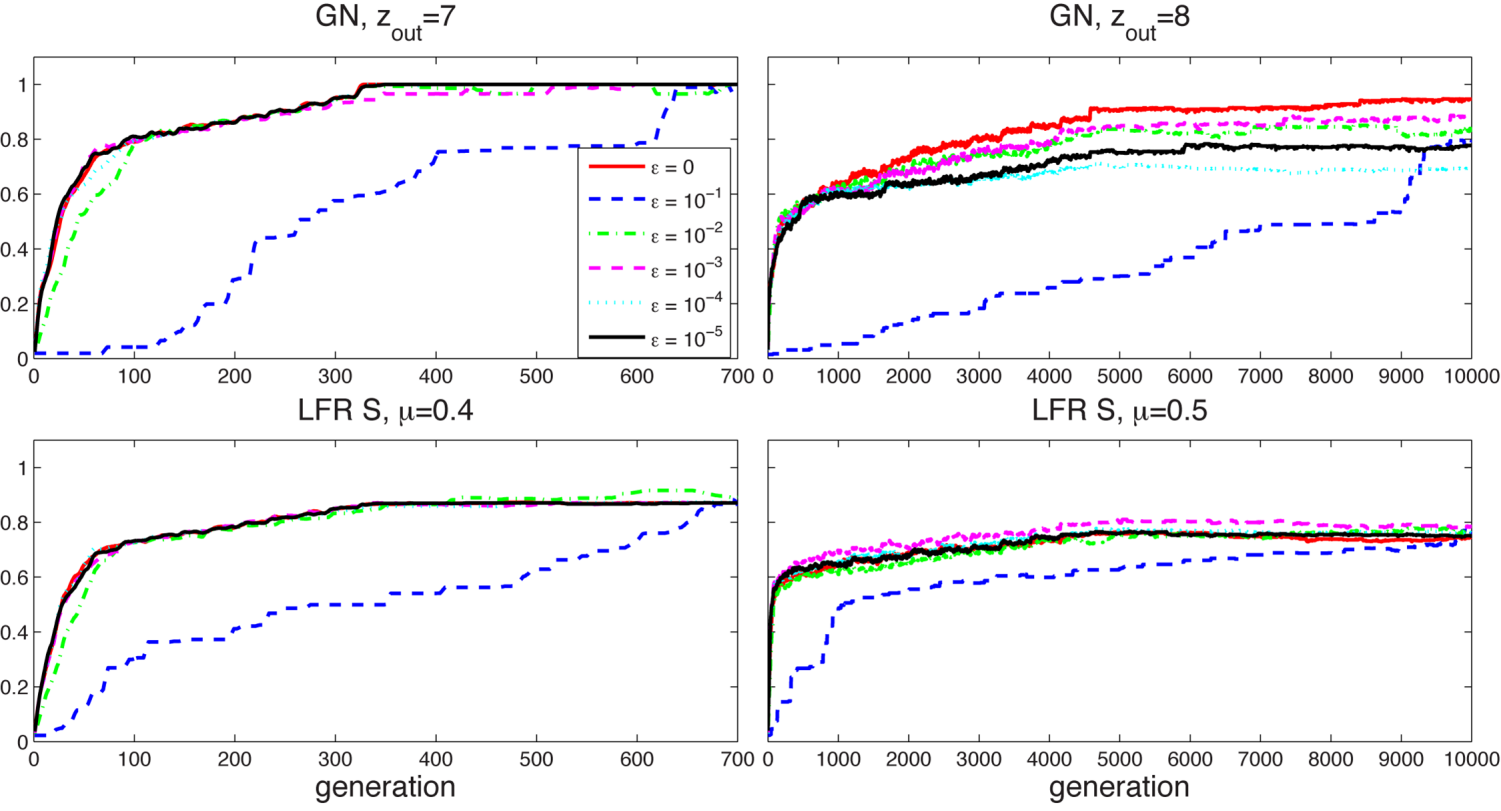
NMI evolution. NMI evolution for GN networks with *z*_*out*_ ∈ {7,8} and LFR S networks with *μ* ∈ {0.4,0.5} for different values of *ϵ*.

A common conclusion that can be drawn from these results is that the value of *ϵ* does not affect the search results if set less than 0.01. Furthermore, Fig 5 illustrates the evolution of NMI values for some of the more difficult synthetic networks and for different values of *ϵ*, showing a distinct -not so good—behavior for *ϵ* = 0.1 and similar trends for all other values.

**Fig 6:**
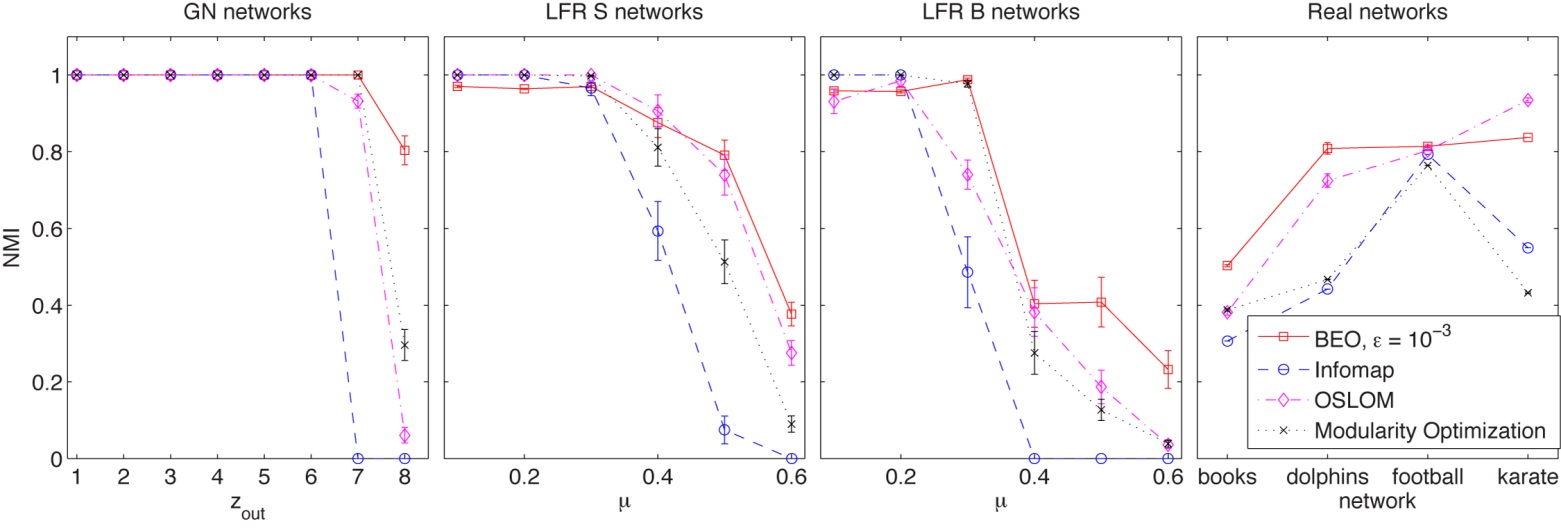
Comparisons. Mean and standard error of the mean of NMI for BEO and the other algorithms for GN, LFR and real networks.

Fig 6 presents mean and standard errors for the results reported by BEO (*ϵ* = 0.001) and Infomap, OSLOM, and Modularity Optimization in order to assess if these results are in fact comparable with those obtained by other methods.

#### Statistical analysis

According to the Wilcoxon sum rank test, BEO results are significantly better than all other methods considered for the GN *z*_*out*_ = 8, LFR small and big for *μ* = 0.5 and 0.6, and for the books and dolphins network. For GN *z*_*out*_ = 7, BEO results are significantly better than those of Oslom and Infomap, and for GN *z*_*out*_ = 6, better than Infomap. For both LFR small and big, *μ* = 0.4 and for the karate network, BEO results are better than those obtained by Infomap and Modularity Optimization. The Wilcoxon sum rank test was performed for all tested values of *ϵ*.

These results indicate the potential of this approach: they show that the concept of *ϵ*-Berge-Zhukovskii equilibrium may be used to approach the community structure detection problem with results just as good and even better than those provided by other state-of-art methods. Many implications arise from here, of both practical and theoretical nature. From a practical point of view, further experiments should be performed to study the behavior of search methods that attempt to compute the Berge-Zhukovskii equilibrium for the community structure detection game. From a theoretical point of view the challenge is to prove that the Berge-Zhukovskii equilibrium of this game indeed represents a network cover.

## Conclusions

Berge-Zhukovskii equilibrium is a powerful concept in game theory, with known applications in trust games. Introduced in this paper, the *ϵ*-Berge-Zhukovskii equilibrium represents a flexible concept that approximates the BZ equilibrium, allowing for a deviation of *ϵ* in payoffs. Thus, it may be also considered as a relaxation of the BZ equilibrium. The set of *ϵ*-Berge-Zhukovskii is characterized by a generative relation.

Apart defining this equilibrium concept, a computational method to approach it is presented. Based on the idea that the equilibrium search is equivalent to the search for nondominated solutions with respect to the generative relation, an evolutionary algorithm for multiobjective optimization is adapted for *ϵ*-Berge-Zhukovskii equilibria detection for the voluntary contribution mechanism game.

The *ϵ*-BZ equilibrium concept is also successfully used to approach the community structure detection problem. This novel application of BZ offers new perspectives over the practical value of this less known equilibrium concept, opening several possibilities for further development.
